# Women’s Health: Mining for Glyceollins

**DOI:** 10.1289/ehp.115-a189a

**Published:** 2007-04

**Authors:** M. Nathaniel Mead

In recent years, agricultural scientists have begun to explore novel strategies for isolating plant compounds of potential medicinal value. One such strategy involves exploiting the defense mechanisms of the plant in order to elicit the production of novel antimicrobial compounds called phytoalexins, which the plants synthesize in response to infection, freezing, and other forms of stress. A recent study spearheaded by researchers at the Tulane Cancer Center and Center for Bioenvironmental Research demonstrates that glyceollins, the main phytoalexins produced by soybeans, may prove to be highly effective in blocking the growth and spread of gynecologic cancers.

Glyceollins are metabolites of the well-studied isoflavone daidzein, and they appear to have more potent bioactivity than the isoflavones in standard soy protein. In work published 1 December 2006 in *Clinical Cancer Research*, the researchers used a procedure developed at the USDA’s Southern Regional Research Center in New Orleans to isolate a mixture of glyceollins I, II, and III. The compounds were extracted from newly germinated soybeans that had been challenged with the food-safe fungus *Aspergillus sojae*.

Previous research by the same team, published in the April 2001 issue of the *Journal of Clinical Endocrinology & Metabolism*, had shown that glyceollins had a marked antiestrogenic effect on estrogen receptor signaling. The new study focused on the effect of the glyceollin mixture on the growth of human estrogen-dependent MCF-7 breast cancer cells and BG-1 ovarian cancer cells. Malignant cells were grafted onto female ovariectomized mice, which were then divided into four treatment groups: control, estradiol only, glyceollins only, and estradiol plus glyceollins. All doses were 20 mg/kg/day.

The researchers report that the glyceollins suppressed MCF-7 tumor growth by 53.4% and BG-1 tumor growth by 73.1%, compared to estradiol alone. In addition, the glyceollins completely suppressed estradiol-induced expression of progesterone receptors in MCF-7 cells and partially suppressed their expression in the BG-1 cells. Thus, glyceollins seem to exert their anticancer activity, in part, by interfering with the cancer cells’ ability to respond to estradiol, the most potent endogenous estrogen and a major growth stimulus for breast and ovarian cancers.

Tulane cancer researcher and report coauthor Matthew Burow asserts that the compounds may eventually play a role in breast cancer prevention and treatment. “Of particular interest is the fact that glyceollins seem to be acting like pure antiestrogens,” he notes. “They show antiestrogen effects in the absence of significant estrogenic or uterotropic activity. This distinguishes them from other phytoestrogens and more importantly from the antiestrogen drug tamoxifen, which is widely used [for breast cancer control] but has been linked with increased risk of uterine cancers.”

Burow adds that some women resist using tamoxifen and other antiestrogenic drugs because of the potential side effects, and for this reason the chemopreventive potential of glyceollin-enriched soy merits further study. He cites a study that he and colleagues from Wake Forest University published in volume 56, issue 1 (2006) of *Nutrition and Cancer*, which found that glyceollin-enriched soy protein had anti-estrogenic effects on normal breast tissue in postmenopausal female monkeys.

Another medical application that may stand to benefit from glyceollin research is hormone replacement therapy, which remains the most effective means for staving off menopausal symptoms and chronic diseases such as osteoporosis. However, hormone replacement therapy carries with it a small increase in breast cancer risk. “The Tulane findings, coupled with the findings from our recent primate study, point to the possibility that the co-administration of soy enriched with glyceollins might diminish the breast cancer risk associated with hormone replacement therapy for peri- and postmenopausal women,” says Thomas B. Clarkson, a professor of comparative medicine at Wake Forest University.

The Tulane research has illuminated new opportunities for both drug development and a greater understanding of environmental influences on biological systems. “This study is a fascinating example of the potential for mining the biochemistry of plants,” says J. Mark Cline, a pathology professor at Wake Forest University who collaborated with Burow and Clarkson on the primate study. “It tells us that we should be paying attention not only to diversity between plant species, but also to biochemical differences within [the same] type of plant under different conditions of culture or stress.”

